# A randomized, phase II study of gefitinib alone versus nimotuzumab plus gefitinib after platinum-based chemotherapy in advanced non-small cell lung cancer (KCSG LU12-01)

**DOI:** 10.18632/oncotarget.13056

**Published:** 2016-11-03

**Authors:** Hye Ryun Kim, Joung Soon Jang, Jong-Mu Sun, Myung-Ju Ahn, Dong-Wan Kim, Inkyung Jung, Ki Hyeong Lee, Joo-Hang Kim, Dae Ho Lee, Sang-We Kim, Byoung Chul Cho

**Affiliations:** ^1^ Department of Internal Medicine, Division of Medical Oncology, Yonsei Cancer Center, Yonsei University College of Medicine, Seoul, Korea; ^2^ Department of Internal Medicine, Chung-Ang University, College of Medicine, Seoul, Korea; ^3^ Department of Medicine, Division of Hematology-Oncology, Samsung Medical Center, Sungkyunkwan University School of Medicine, Seoul, Korea; ^4^ Department of Internal Medicine, Seoul National University Hospital, Seoul, Republic of Korea; ^5^ Department of Biostatistics and Medical Informatics, Yonsei University College of Medicine, Seoul, Korea; ^6^ Department of Internal Medicine, Chungbuk National University, College of Medicine, Cheongju, Korea; ^7^ Department of Oncology, University of Ulsan College of Medicine, Asan Medical Center, Seoul, Korea

**Keywords:** non-small cell lung cancer, epidermal growth factor receptor, nimotuzumab, gefitinib

## Abstract

We aimed to evaluate the efficacy of dual inhibition of epidermal growth factor receptor (EGFR) with nimotuzumab (EGFR monoclonal antibody) plus gefitinib (EGFR-tyrosine kinase inhibitor) in advanced non-small cell lung cancer (NSCLC) after platinum-based chemotherapy. An open label, randomized, phase II trial was conducted at 6 centers; 160 patients were randomized (1:1) to either gefitinib alone or nimotuzumab (200 mg, i.v. weekly) plus gefitinib (250 mg p.o. daily) until disease progression or intolerable toxicity. The primary endpoint was progression-free survival (PFS) at 3 months. Of the total 160 enrolled patients, 155 (77: gefitinib, 78: nimotuzumab plus gefitinib) received at least one dose and could be evaluated for efficacy and toxicity. The majority had adenocarcinoma (65.2%) and ECOG performance status of 0 to 1 (83.5%). The median follow-up was 22.1 months, and the PFS rate at 3 months was 48.1% in gefitinib and 37.2% in nimotuzumab plus gefitinib (*P* = not significant, NS). The median PFS and OS were 2.8 and 13.2 months in gefitinib and 2.0 and 14.0 months in nimotuzumab plus gefitinib. Combined treatment was not associated with superior PFS to gefitinib alone in patients with *EGFR* mutation (13.5 *vs*. 10.2 months in gefitinib alone, *P*=NS) or those with wild-type *EGFR* (0.9 *vs*. 2.0 months in gefitinib alone, *P*=NS). Combined treatment did not increase EGFR inhibition-related adverse events with manageable toxicities. The dual inhibition of EGFR with nimotuzumab plus gefitinib was not associated with better outcomes than gefitinib alone as a second-line treatment of advanced NSCLC (NCT01498562).

## INTRODUCTION

Lung cancer is the second most common cancer type and the leading cause of cancer death worldwide [1, 2]. Non-small cell lung cancer (NSCLC) is approximately 85% of lung malignancies and most patients are diagnosed with far advanced stage NSCLC. Although agents that target specific genetic alterations have been developed for lung cancer treatment, the prognosis of lung cancer is still poor and the 5-year survival rate has been less than 5% in advanced-stage disease.

The epidermal growth factor receptor (EGFR) is overexpressed on various types of solid cancers, including NSCLC [3]. Although the prognostic or predictive function of EGFR expression in patients with NSCLC remains controversial, some researches have suggested that upregulation of EGFR expression is related to tumor establishment and spread and poor prognosis in NSCLC [3–5]. Dysregulation of EGFR in several tumors including lung cancer, head and neck cancer, esophageal cancer, and others, correlated with metastasis and poor survival outcome [5]. Increased knowledge of EGFR signaling pathway regulation in NSCLC has led to develop targeted agents. Anti-EGFR monoclonal antibodies which bind to the extracellular domain of this receptor, or small-molecule tyrosine kinase inhibitors which block the kinase domain are currently available EGFR-targeted drugs [3, 6, 7]. These EGFR-targeted agents have been related with improvement in survival of NSCLC patients. Recently, the SQUIRE, FLEX, and INSPIRE trials showed that the combination of EGFR monoclonal antibodies, such as necitumumab and cetuximab, with platinum-based cytotoxic chemotherapy as a first-line therapy in squamous cell lung cancer led to prolonged survival outcomes, while the same results were not seen in adenocarcinoma [8–11]. However, the role of anti-EGFR antibodies in NSCLC has not been established.

Nimotuzumab (TheraCIM^®^) is a humanized IgG1 mAb targeting EGFR [3]. Nimotuzumab adheres to the extracellular domain III of EGFR with moderate affinity, blocking EGF ligand binding and receptor dimerization by steric hindrance [3]. Nimotuzumab demonstrated inhibitory activity on tumor cell growth, angiogenesis, and apoptosis [5, 12]. Phase I and II clinical trials with nimotuzumab presented the lack of a severe dermatologic reaction unlike other anti-EGFR drugs [3, 13]. In previous trials that focused primarily on brain malignancies or head and neck cancer, nimotuzumab has demonstrated greater efficacy similar to other anti-EGFR mAbs [3, 14–17]. Two recent phase I clinical trials in NSCLC patients demonstrated the minimal toxicity of nimotuzumab combined with radiation therapy and also presented favorable antitumor effects [3, 18, 19].

Activating mutations of the *EGFR* gene, mainly exon 19 deletions and exon 21 a single missense mutation, were known to correlate with clinical response to gefitinib [3]. Gefitinib significantly improve progression-free survival (PFS) in NSCLC patients who had activating *EGFR* mutations. Three phase III clinical trials conducted exclusively in *EGFR* mutated patients [6, 7, 20] demonstrated that patients with *EGFR* mutations experienced longer PFS with gefitinib treatment than cytotoxic chemotherapy. These large-scale trials suggested that *EGFR* mutation in tumors is a strong predictive biomarker of better gefitinib-associated outcomes.

Many preclinical data support the combination of EGFR targeting therapy with EGFR-TKIs and anti-EGFR mAbs to get over intrinsic or acquired resistance mechanisms and to amplify the potency of EGFR signaling inhibition[3, 21–23]. These data suggest that dual blockade of EGFR pathway could be new treatment strategies to maximize effective target inhibition. Recent phase I/II trials that added cetuximab to erlotinib demonstrated stable disease in majority of patients (11 of 13) with acquired resistance, although there was no partial response on radiologic images. It is to be noted that one third of the patients discontinued combined therapy due to intolerable rash [3, 24]. Thus, it is important to develop a tolerable dual blockade of the *EGFR* pathway with EGFR mAb plus EGFR-TKI and to identify clinical applicability.

We already performed the phase I clinical trial to determine recommended phase II dosing (RPIID) and safety of the combination of gefitinib and nimotuzumab [3]. This phase I trial demonstrated a well-tolerated safety profile without dose-limiting toxicity and 25% partial response (4/16 patients) and 43.8% stable disease (7/16 patients) [3]. Based on these phase I trial results, the RPIID for nimotuzumab is a 200 mg weekly i.v. and for gefitinib 250 mg daily p.o.[3] We evaluated the efficacy of dual inhibition of the EGFR pathway with nimotuzumab plus gefitinib in advanced NSCLC patients who were previously treated with platinum-based chemotherapy.

## RESULTS

### Patient characteristics

**Figure 1:**
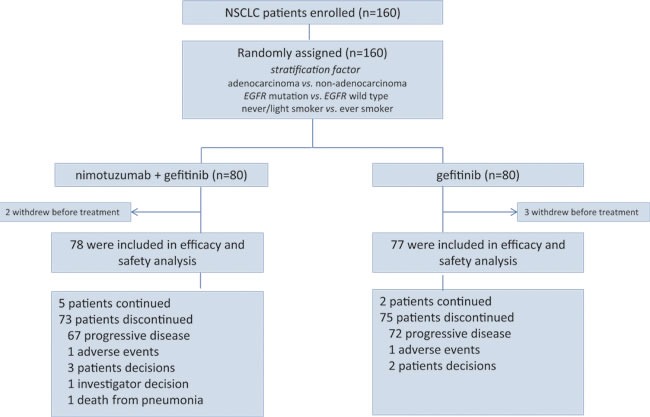
Trial profile in this study

A total of 160 patients were enrolled between Feb. 2012 and Jun. 2014 at 6 centers in Korea. Of 160 patients, 80 were randomly allocated to the gefitinib and 80 patients were assigned to nimotuzumab plus gefitinib. Five patients (2 in nimotuzumab plus gefitinib 3 in gefitinib arm) withdrew consent before treatment. Finally, 155 patients received at least one dose and could be evaluated for efficacy and toxicity in this study. Progressive disease was confirmed in 139 patients by the data cutoff point of March, 30^th^ 2016 (Figure 1).

The median age was 63 years old and 102 patients (63.8%) were male. The majority (98.1%) were ECOG PS 0 or 1. Fifty-six patients (35.0%) were never/light smokers and 106 patients (66.2%) had adenocarcinoma. The stratification factors were well balanced in nimotuzumab plus gefitinib and gefitinib groups: 66.2% and 66.2% in adenocarcinoma, 13.8% and. 25.0% in *EGFR* mutation, and 31.2% and 38.8% in never smoker. Patient characteristics were well assigned in both groups and patient baseline characteristics were summarized in Table 1.

**Table 1:** Baseline patient characteristics

Characteristics	Total (*n* = 160)	Experimental arm Nimotuzumab+ Gefitinib (*n* = 80)	Control arm Gefitinib (*n* = 80)	*P*
	***n*** **(%)**	***n*** **(%)**	***n*** **(%)**	
*Age, years*				0.80
Median	63	63	63	
Range	31-84	31-84	37-83	
*Sex*				0.41
Male	102 (63.8%)	54 (67.5%)	48 (60.0%)	
Female	58 (36.2%)	26 (32.5%)	32 (40.0%)	
*ECOG performance status*				0.13
0	77 (49.7%)	41 (52.6%)	36 (46.8%)	
1	75 (48.4%)	34 (43.6%)	41 (53.2%)	
2	3 (1.9%)	3(3.8%)	0 (0%)	
*Smoking*				0.40
Never/light smoker	56 (35.0%)	25 (31.2%)	31 (38.8%)	
Ever smoker	104 (65%)	55 (68.8%)	49 (61.2%)	
*Histology*				0.56
Adenocarcinoma	106 (66.2%)	53 (66.2%)	53 (66.2%)	
Non-adenocarcinoma	54 (33.8%)	27 (33.8%)	27 (33.8%)	
*Brain metastasis*				0.65
Yes	30 (18.8%)	16 (20.0%)	14 (17.5%)	
*EGFR mutation*				0.36
Positive	31 (19.4%)	11 (13.8%)	20 (25.0%)	
Exon19 del	14 (8.8%)	3 (3.8%)	11 (13.8%)	
Exon21 L858R	11 (6.9%)	7 (8.8%)	4 (5.0%)	
Other^†^	6 (3.8%)	1 (1.2%)	5 (6.3%)	
Negative	84 (52.5%)	39 (48.8%)	38 (47.5%)	
Not evaluable	45 (26.9%)	29 (36.2%)	27 (33.8%)	
*KRAS mutation*				0.59
Positive	7 (4.3%)	5 (6.2%)	2 (2.5%)	
Negative	70 (43.8%)	36 (45.0%)	34 (42.5%)	
Unknown	83 (51.9%)	39 (48.8%)	44 (55.0%)	
Previous first-line therapy				0.68
Platinum + paclitaxel	77 (49.0%)	38 (48.1%)	39 (50.0%)	
Platinum + gemcitabine	41 (26.1%)	22 (27.8%)	19 (24.4%)	
Platinum + pemetrexed	35 (22.3%)	16 (20.3%)	19 (24.4%)	
Other	4 (2.5%)	3 (3.8%)	1 (1.3%)	

Abbreviations: ECOG, Eastern Cooperative Oncology Group; EGFR, epidermal growth factor receptor; †These six patients had in three exon 18 mutation (G719X) and three exon20 insertion mutation.

Of 160 enrolled patients, 115 were available for *EGFR* mutation analysis, mainly because of insufficient tissue materials. Among 115 with available results of *EGFR* mutation, *EGFR* mutation (14 in Exon 19 del mutation, 11 in Exon21 L858R mutation, 3 in Exon18 G719X mutation and 3 in Exon 20 insertion mutation) was documented in 31 patients. Eleven of these patients were in nimotuzumab plus gefitinib and 20 patients in gefitinib alone. Seventy-seven patients were available for *KRAS* mutation results and 7 (4.3%) patients were positive (5 patients in nimotuzumab plus gefitinib and 2 patients in gefitinib). In regards to previous platinum based chemotherapy regimen, platinum + paclitaxel was performed in 49.0% of patients, platinum + gemcitabine in 26.1%, and platinum + pemetrexed in 22.3%.

### Clinical outcomes

**Figure 2:**
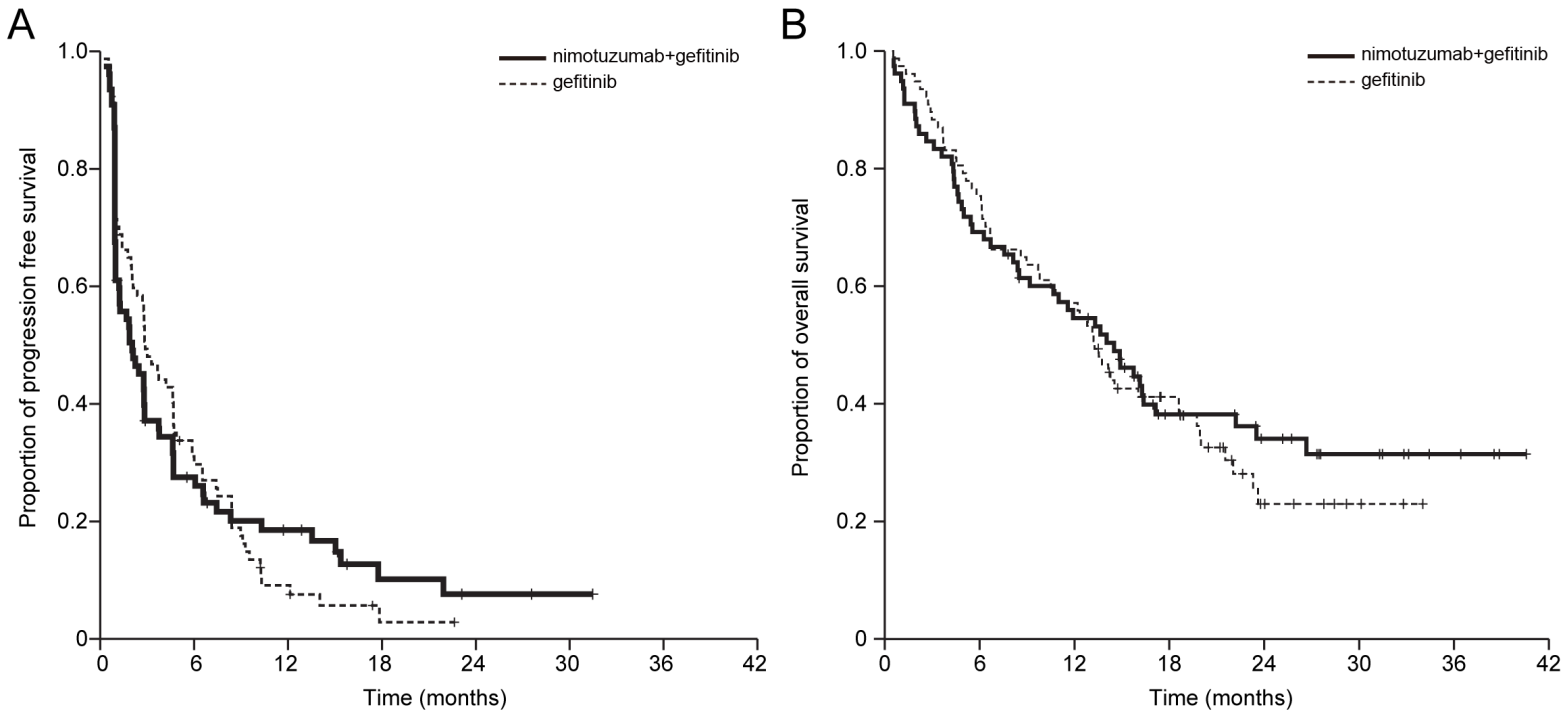
Kaplan-Meier estimates of **A**. progression-free survival (PFS) and **B**. overall survival (OS) of patients with advanced non-small cell lung cancer that were treated. The differences in median PFS (HR 1.03, 95% CI 0.71-1.41, *P* = 0.98) and OS (HR 0.86, 95% CI 0.57–1.30) were not statistically significant between the nimotuzumab plus gefitinib and the gefitinib arm.

The median duration of nimotuzumab plus gefitinib and gefitinib administration was 1.8 (range, 0.2-31.5 months) and 2.8 (range, 0.1-22.6 months) months. The ORRs were 22.1% in gefitinib arm and 16.7% in nimotuzumab plus gefitinib arm, without statistical significance (*P* = 0.35) (Supplementary Figure 1, Table 2). With a median follow-up duration of 22.1 months, the PFS rate at 3 months was 37.2% in nimotuzumab plus gefitinib and 48.1% in gefitinib [HR 0.99; 95% CI, 0.70-1.38; *P* = 0.95]. The median PFS and OS were 2.0 months and 14.5 months in nimotuzumab plus gefitinib and 2.8 months and 13.2 months in gefitinib [HR 0.99; 95% CI, 0.70-1.38; *P* = 0.95 for PFS; HR 0.93, 95% CI 0.63-1.38, *P* = 0.72 for OS] (Figure 2).

**Table 2:** Tumor response according to RECIST

Response	Total† (*n* = 155)	Nimotuzumab+ Gefitinib (*n* = 78)	Gefitinib (*n*= 77)	*P*
	***n*** **(%)**	***n*** **(%)**	***n*** **(%)**	
Response				0.27
CR	0 (0%)	0 (0%)	0 (0%)	
PR	30 (19.4%)	13 (16.7%)	17 (22.1%)	
SD	62 (40.0%)	29 (37.2%)	33 (42.9%)	
PD	57 (36.8%)	31 (39.7%)	26 (33.8%)	
Not assessable	6 (3.9%)	5 (6.4%)	1 (1.3%)	
Overall response rate*, %	30 (19.4%)	13 (16.7%)	17 (22.1%)	0.35
Disease control rate§, %	92 (59.4%)	42 (53.9%)	50 (64.9%)	0.54
PFS (month)				
Median (95%, CI)	2.7 (2.1-3.3)	2.0 (0.7-3.3)	2.8 (1.9-3.7)	0.95
OS (month)				
Median (95%, CI)	13.7 (11.1-16.3)	14.0 (9.7-18.2)	13.5 (11.3-15.7)	0.72

† Five patients (2 in the experimental and 3 in the control arm) withdrew from study before treatment, and were excluded from analysis.

Tumor responses were assessed with the use of Response Criteria in Solid Tumors (RECIST), version 1.1

§The disease control rate was calculated as complete response plus partial response plus stable disease

Abbrviations: RECIST, Response Evaluation Criteria in Solid Tumors; CR, complete response; PR, partial response; SD, stable disease; PD, progressive disease; PFS, progression free survival; OS, overall survival; CI, confidence interval

**Figure 3:**
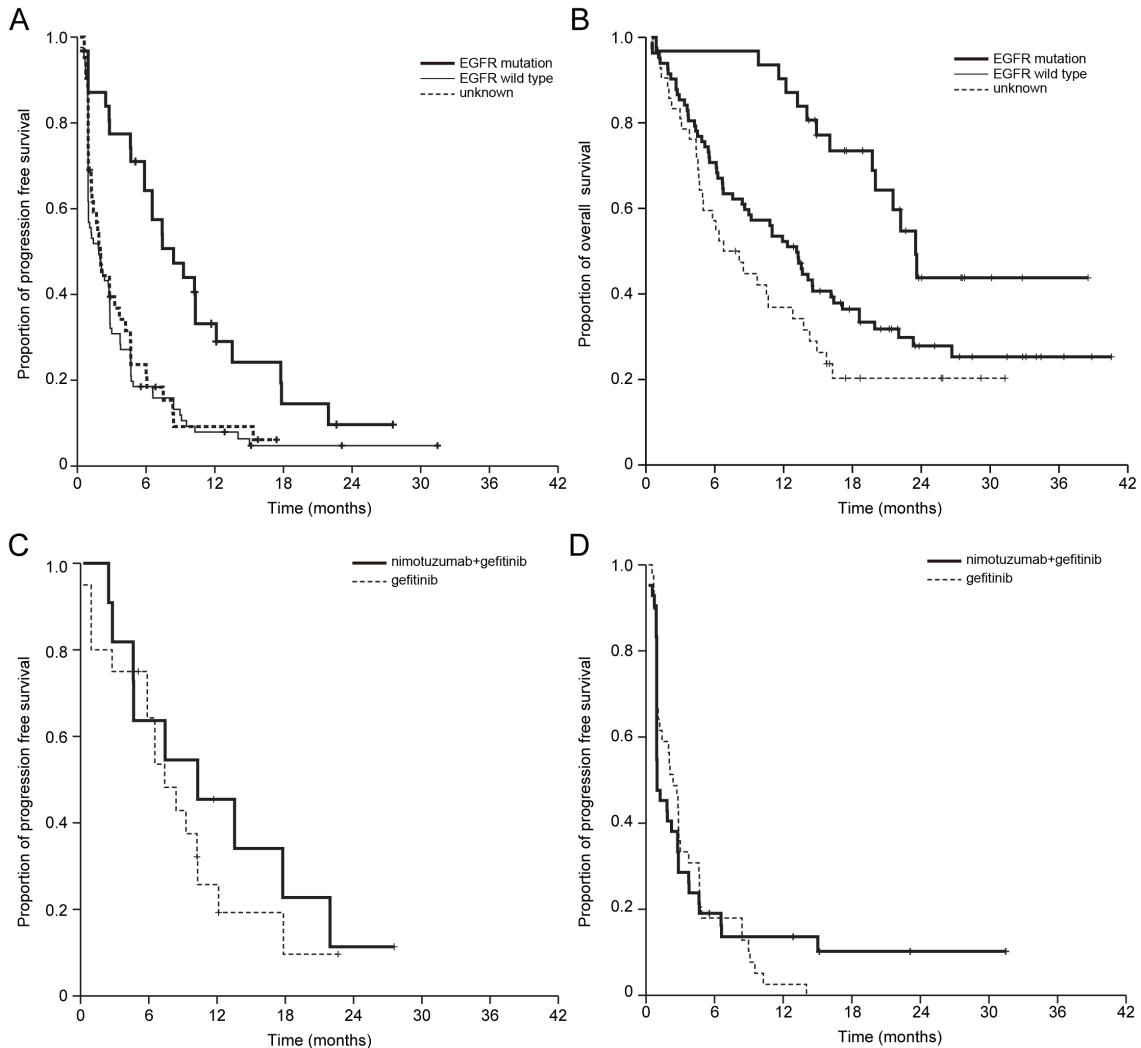
Kaplan-Meier estimates of **A**. progression-free survival (PFS) and **B**. overall survival (OS) of all patients according to *EGFR* mutation status. Patients with *EGFR* mutations showed significantly longer survival than those with wild-type *EGFR* or unknown *EGFR* mutation statuses (8.4 vs. 1.8 *vs*. 2.0 months, *P* < 0.001 for PFS; 23.5 vs. 13.1 *vs*. 6.7 months, *P* = 0.001 for OS). Nimotuzumab plus gefitinib was not found to have superior PFS compared with gefitinib monotherapy **C**. in patients with *EGFR* mutations (10.3 *vs*. 7.4 months in gefitinib alone, *P* = 0.42) or **D**. wild type EGFR patients (1.0 *vs*. 2.3 months in gefitinib alone, *P* = 0.85).

As expected, patients who had *EGFR* mutations demonstrated significantly longer survival than those with wild-type *EGFR* or unknown *EGFR* mutation (8.4 *vs*. 1.8 *vs*. 2.0 months, *P* < 0.001 for PFS; 23.5 *vs*. 13.1 *vs*. 6.7 months, *P* = 0.001 for OS) (Figure 3A and 3B). Combined treatment of nimotuzumab plus gefitinib was not superior in PFS compared to gefitinib alone in patients with *EGFR* mutations (10.3 *vs*. 7.4 months in gefitinib alone, *P* = 0.42) and patients with wild-type *EGFR* (1.0 *vs*. 2.3 months in gefitinib alone, *P* = 0.85) (Figure 3C and 3D). The histology results indicated that median PFS was not significantly different between the two treatment arms (2.8 *vs*. 2.9 months in gefitinib alone for adenocarcinoma, *P* = 0.64; 1.2 *vs*. 2.8 months in gefitinib alone for non-adenocarcinoma, *P* = 0.35). Altogether, the dual inhibition of EGFR with nimotuzumab plus gefitinib was not associated with better outcomes compared with gefitinib alone for second-line therapy of advanced NSCLC.[3]

### Safety

All patients who had received at least one dose of a study drug were analyzed in the safety analysis. Adverse events (AEs) in both treatment arms were mostly grade 1 to 2 and easily manageable. Importantly, combined EGFR inhibition with nimotuzumab and gefitinib did not increase EGFR inhibition-related AEs, such as acneiform rash (47.4% *vs*. 44.9% in gefitinib alone, *P* = 0.40), diarrhea (35.9 *vs*. 35.1% in gefitinib alone, *P* = 0.52), and stomatitis (23.1 *vs*. 26.0% in gefitinib alone, *P* = 0.40) (Table 3). Treatment-related death was not occurred in current trial.

**Table 3:** Treatment-related adverse events (*N* = 155)†

	Nimotuzumab+Gefitinib (*n*=78)			Gefitinib (*n*=75)			*P*-value
**Adverse event**	**All grades**	**Grade 1-2**	**Grade≥3**	**All grades**	**Grade 1-2**	**Grade≥3**	
	***N*** **(%)**			**N (%)**			
Acneiform rash	37(47.4%)	35(44.9%)	2 (2.6%)	34(44.2%)	33(42.9%)	1 (1.3%)	0.40
Diarrhea	28 (35.9%)	26 (33.3%)	2 (2.6%)	27 (35.1%)	26 (33.8%)	1 (1.3%)	0.52
Anorexia	27 (34.6%)	25 (32.1%)	2 (2.6%)	21 (27.3%)	18 (23.4%)	3 (3.9%)	0.28
Stomatitis	18 (23.1%)	18 (23.1%)	0 (0%)	20 (26.0%)	20 (26.0%)	0 (0%)	0.40
Dry skin	16 (20.5%)	16 (20.5%)	0 (0%)	16 (20.5%)	16 (20.5%)	0 (0%)	0.40
Pruritus	16 (20.5%)	16 (20.5%)	0 (0%)	16 (20.8%)	16 (20.8%)	0 (0%)	0.56
Paronychia	12 (15.4%)	12 (15.4%)	0 (0%)	13 (16.9%)	13 (16.9%)	0 (0%)	0.48
AST elevation	11(14.1%)	9 (11.5%)	2 (2.6%)	8 (10.4%)	6 (7.8%)	2 (2.6%)	0.32
ALT elevation	10 (12.8%)	8 (10.3%)	2 (2.6%)	8 (10.4%)	5 (6.5%)	3 (3.9%)	0.29
Myalgia	9 (11.6%)	9 (11.6%)	0 (0%)	7 (9.1%)	7 (9.1%)	0 (0%)	0.60
Nausea	8 (10.3%)	8 (10.3%)	0 (0%)	10 (13.0%)	8 (10.4%)	2 (2.6%)	0.39
Vomiting	5 (6.4%)	5 (6.4%)	0 (0%)	4 (5.2%)	4 (5.2%)	0 (0%)	0.50
Thrombocytopenia	5 (6.4%)	5 (6.4%)	1 (1.3%)	3 (3.9%)	3 (3.9%)	0 (0%)	0.56
Neutropenia	4 (5.1%)	4 (5.1%)	0 (0%)	2 (3.9%)	2 (3.9%)	0 (0%)	0.50
Anemia	3 (3.8%)	3 (3.8%)	0 (0%)	4(5.2%)	4(5.2%)	0 (0%)	0.49
Pneumonitis	3 (3.8%)	3 (3.8%)	0 (0%)	3 (3.9%)	2 (2.6%)	1 (1.3%)	0.65

Toxic-effect grades are based on the National Cancer Institute Common Terminology Criteria (version 3.0).

## DISCUSSION

This is the first randomized phase II clinical trial that assessed combination therapy of nimotuzumab plus gefitinib compared to gefitinib in advanced NSCLC patients who failed to platinum-based chemotherapy. This study presented the feasibility and tolerability of combination therapy with nimotuzumab and gefitinib in advanced lung cancer patients. However, this randomized phase II clinical trial did not prove its primary endpoint, as the PFS rate at 3 months was 37.2% in the nimotuzumab plus gefitinib compared to 48.1% in the gefitinib alone. Moreover, in both patients with and without *EGFR* mutation, combined nimotuzumab plus gefitinib did not demonstrate favorable PFS outcomes compared to gefitinib alone. Collectively, this study demonstrated that the dual inhibition of EGFR with nimotuzumab plus gefitinib was not superior to gefitinib alone as second-line treatment of advanced NSCLC patients. Combined therapy with nimotuzumab plus gefitinib showed a manageable toxicity profile, with rash and diarrhea as the most frequent treatment-related AEs. The safety profile for the combination of nimotuzumab and gefitinib was comparable with that of each individual drug and any unexpected toxicities were not developed.

Our hypothesis was that dual blockade of the EGFR pathway using a combination of nimotuzumab plus gefitinib could improve survival outcomes in advanced NSCLC patients. However, dual blockade of EGFR pathway did not demonstrate favorable survival benefit compared to gefitinib alone regardless of *EGFR* mutation status. Although the numbers of *EGFR* mutated patients in this study were too small to make conclusion, dual blockade of the *EGFR* pathway might not be associated with superior treatment outcomes over the monotherapy with gefitinib even in EGFR mutated patients as well as EGFR wild type.

In contrast to previous studies, in our study, treatment with the EGFR monoclonal antibody nimotuzumab did not prolong survival in squamous cell lung cancer patients. In patients who had acquired resistance to EGFR-TKI, dual blockage of EGFR with afatinib and cetuximab demonstrated robust clinical activity in both those with and without T790M mutations [25]. This result implicated that a significant portion of tumors in patients with acquired resistance to gefitinib or erlotinib still remain dependent on EGFR signaling for survival. Therefore, more complete blockade of EGFR is an effective strategy that could be worthwhile for further study in *EGFR* mutated patients with acquired resistance.

There are several potential reasons for the lack of variation in outcomes between combined- and mono-therapy. Firstly, the affinity of nimotuzumab is ~10 times lower than those of cetuximab and necitumumab, and thus this lower affinity of nimotuzumab might cause the negligible blocking effect of the EGFR pathway seen in our study [13]. Additionally, the small number of enrolled patients, slight imbalance in prognostic factors between the two treatment arms favoring the gefitinib arm, and lack of patient selection based on EGFR expression could have led to the negative findings. The percentage of *EGFR* mutation was quite high in the gefitinib arm; although this difference was not statistically significant, it may have had an impact on treatment outcomes in this clinical trial.

With regard to AEs, nimotuzumab did not cause severe dermatologic reactions compared to other anti-EGFR antibodies [9, 24, 25]. Combined EGFR inhibition with nimotuzumab and gefitinib also did not increase EGFR inhibition-related AEs. Considering this favorable toxic profile, nimotuzumab could be a good candidate anti-EGFR antibody when combined with other drugs.

Conclusively, the dual inhibition of EGFR pathway with nimotuzumab plus gefitinib was not associated with better outcomes compared with gefitinib alone for second-line treatment of advanced NSCLC patients, regardless of *EGFR* mutation status.

## PATIENTS AND METHODS

### Study design

An open label, randomized, phase II trial was designed to prospectively evaluate the efficacy of dual inhibition of EGFR with nimotuzumab plus gefitinib in advanced NSCLC patients that were previously treated with platinum-based chemotherapy. A total of 160 patients were allocated in a 1:1 ratio to either the treatment arm (nimotuzumab plus gefitinib) or the control arm (gefitinib alone) using the minimization method to balance to center, histology (adenocarcinoma vs. non-adenocarcinoma), *EGFR* mutation (*EGFR* mutation *vs*. wild type), and smoking status (never/light *vs*. ever smoker). The treatment arm consisted of 200 mg of nimotuzumab administered via intravenous infusion over 30 min, once per week, and gefitinib at a fixed dose of 250 mg daily until disease progression or unacceptable toxicity. The control arm received gefitinib at a fixed dose of 250 mg daily. Each treatment cycle was defined as 28 days, regardless of omitted doses. Further treatment after disease progression was at the physician's discretion.

The primary endpoint was the evaluation of the PFS rate at 3 months. Secondary endpoints included PFS, OS, ORR and safety. PFS was assessed from the date of randomization to that of disease progression determined by CT or MRI using RECIST criteria or death from any cause. OS was assessed from the date of randomization until death from any cause. Target lesions were assessed via an independent central review at baseline (within 4 weeks of randomization). To assess toxicity, the National Cancer Institute Common Toxicity Criteria (version 4.0) were used during the study period and follow-up.

This study was conducted in accordance with the Declaration of Helsinki, the International Conference on Harmonization Guidelines on Good Clinical Practice. The protocol was approved by the Protocol Review Committee of the Korean Cancer Study Group and by the Institutional Review Board at each participating institute. All patients provided written informed consent. Study protocol and informed consent forms were approved by institutional review boards of each institution. The trial is registered on ClinicalTrials.gov (clinicaltrials.gov identifier NCT01498562, protocol number: KCSG LU12-01).

### Patient eligibility

Trial eligibility required pathologically or cytologically confirmed stage IV NSCLC; measurable disease by Response Evaluation Criteria in Solid Tumors Group (RECIST); prior systemic therapy with platinum-based chemotherapy; a performance status of 0,1, or 2 based on Eastern Cooperative Oncology Group criteria; age 18 years or older; and adequate bone marrow, liver, and kidney function. Patients with central nervous system metastases were eligible if there were no symptoms or treatment for brain metastasis had been completed. Patients with prior malignancies were eligible if there was no evidence for recurrence for at least 5 years.

### Response evaluation

We performed computed tomography or magnetic resonance imaging scans firstly after 4 weeks, and then every 8 weeks, or at the time when disease progression was suspected. Based on the Response Evaluation Criteria in Solid Tumor (RECIST version 1.1) criteria, responses were assessed and categorized as complete remission (CR), partial response (PR), stable disease (SD) or progressive disease (PD).

### Safety and tolerability

Throughout the study, safety was assessed by monitoring and recording AEs, vital signs, clinical chemistry, hematology, and urinalysis. Event severity was graded according to the National Cancer Institute Common Terminology Criteria Adverse Events version 4.0. Dose modification for gefitinib and nimotuzumab was not allowed. Treatment interruption was required for grade 3 and 4 toxicities per the protocol algorithm. A cycle could be delayed up to 2 weeks to allow sufficient time for recovery. If treatment could not be started after 2 weeks, the patient was removed from the study.

### Statistical analysis

We used the stratified log-rank test for PFS and OS. The ORR and other categorical outcomes between the two groups were compared using the chi-squared test or Fisher's exact test. Multivariable cox regression was performed to adjust for potential confounders such as sex, age, and performance status. We assumed that a PFS rate at 3 months of 0.55 would indicate clinical usefulness of the regimen, whereas a PFS rate at 3 months of 0.4 would be the lower limit of interest. Assuming 12 months for accrual and additional 12 months for follow-up, 71 patients per arm were needed to achieve 80% power to detect a difference of 0.15 in PFS rate at 3 months at a two-sided type. Taking drop-out rate into account, the total sample size was set at 160 (80 for each treatment arm). Efficacy and safety analyses were planned for patients who received at least 1 dose of the treatment. PFS and OS were analyzed using the Kaplan–Meier method to estimate the median values with 95% CIs. Two-sided *P*-values less than 0.1 for the PFS and less than 0.05 for the others were considered statistically significant. All statistical analyses were performed using SPSS ver. 16.0 for Windows.

## SUPPLEMENTARY MATERIALS FIGURE


