# Correction: A randomized, phase II study of gefitinib alone versus nimotuzumab plus gefitinib after platinum-based chemotherapy in advanced non-small cell lung cancer (KCSG LU12-01)

**DOI:** 10.18632/oncotarget.20268

**Published:** 2017-08-14

**Authors:** Hye Ryun Kim, Joung Soon Jang, Jong-Mu Sun, Myung-Ju Ahn, Dong-Wan Kim, Inkyung Jung, Ki Hyeong Lee, Joo-Hang Kim, Dae Ho Lee, Sang-We Kim, Byoung Chul Cho

**Present:** The current funding information is incomplete.

**Correct:** The complete funding information is given below.

Original article: Oncotarget. 2017; 8:15943-15951. https://doi.org/10.18632/oncotarget.13056

