# Supplementary material for: A randomized, phase II study of gefitinib alone versus nimotuzumab plus gefitinib after platinum-based chemotherapy in advanced non-small cell lung cancer (KCSG LU12-01)
Source: Oncotarget. 2016 Nov 3;8(9):15943–51. doi: 10.18632/oncotarget.13056 (PMC5362536; doi:10.18632/oncotarget.13056)
Supplement: Supplementary file 1 [file oncotarget-08-15943-s001.pdf]

A randomized, phase II study of gefitinib alone versus nimotuzumab plus gefitinib after platinum-based chemotherapy in advanced non-small cell lung cancer (KCSG LU12-01)

Supplementary Material

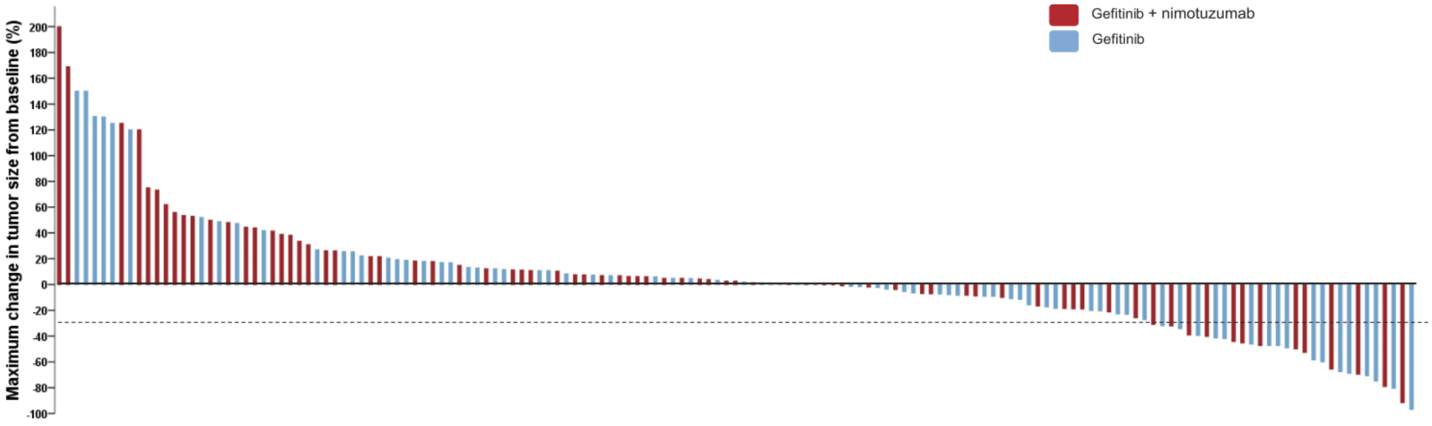

Supplementary Figure 1. Waterfall plot of the maximum change in tumor size from baseline. There was no significant difference in regards to the magnitude of tumor reduction between the control arm and the experimental arm.
